# Sensitivity to Sevoflurane anesthesia is decreased in mice with a congenital deletion of Guanylyl Cyclase-1 alpha

**DOI:** 10.1186/s12871-017-0368-5

**Published:** 2017-06-14

**Authors:** Yasuko Nagasaka, Martin Wepler, Robrecht Thoonen, Patrick Y. Sips, Kaitlin Allen, Jan A. Graw, Vincent Yao, Sara M. Burns, Stefan Muenster, Peter Brouckaert, Keith Miller, Ken Solt, Emmanuel S. Buys, Fumito Ichinose, Warren M. Zapol

**Affiliations:** 1 Department of Anesthesia, Critical Care and Pain Medicine, Massachusetts General Hospital, Harvard Medical School, Boston, MA USA; 2 Department of Medicine, Massachusetts General Hospital, Harvard Medical School, Boston, USA; 3 Department of Medicine, Brigham and Women’s Hospital, Harvard Medical School, Boston, USA; 4 Department of Biomedical Molecular Biology, Ghent University, Ghent, Belgium and Inflammation Research Center, VIB, Ghent, Belgium

**Keywords:** Nitric oxide, Soluble guanylyl cyclase, Knock-out mouse, Volatile anesthetics, Sevoflurane, Righting reflex, Cyclic guanosine monophosphate

## Abstract

**Background:**

Volatile anesthetics increase levels of the neurotransmitter nitric oxide (NO) and the secondary messenger molecule cyclic guanosine monophosphate (cGMP) in the brain. NO activates the enzyme guanylyl cyclase (GC) to produce cGMP. We hypothesized that the NO-GC-cGMP pathway contributes to anesthesia-induced unconsciousness.

**Methods:**

Sevoflurane-induced loss and return of righting reflex (LORR and RORR, respectively) were studied in wild-type mice (WT) and in mice congenitally deficient in the GC-1α subunit (GC-1^−/−^ mice). Spatial distributions of GC-1α and the GC-2α subunit in the brain were visualized by in situ hybridization. Brain cGMP levels were measured in WT and GC-1^−/−^ mice after inhaling oxygen with or without 1.2% sevoflurane for 20 min.

**Results:**

Higher concentrations of sevoflurane were required to induce LORR in GC-1^−/−^ mice than in WT mice (1.5 ± 0.1 vs. 1.1 ± 0.2%, respectively, *n* = 14 and 14, *P* < 0.0001). Similarly, RORR occurred at higher concentrations of sevoflurane in GC-1^−/−^ mice than in WT mice (1.0 ± 0.1 vs. 0.8 ± 0.1%, respectively, *n* = 14 and 14, *P* < 0.0001). Abundant GC-1α and GC-2α mRNA expression was detected in the cerebral cortex, medial habenula, hippocampus, and cerebellum. Inhaling 1.2% sevoflurane for 20 min increased cGMP levels in the brains of WT mice from 2.6 ± 2.0 to 5.5 ± 3.7 pmol/mg protein (*n* = 13 and 10, respectively, *P* = 0.0355) but not in GC-1^−/−^ mice.

**Conclusion:**

Congenital deficiency of GC-1α abolished the ability of sevoflurane anesthesia to increase cGMP levels in the whole brain, and increased the concentration of sevoflurane required to induce LORR. Impaired NO-cGMP signaling raises the threshold for producing sevoflurane-induced unconsciousness in mice.

## Background

Intraoperative awareness is a major problem in anesthesia [1], and it remains unknown why some patients are resistant to anesthesia and more susceptible to experiencing recall after an operation. People with red hair have been reported to require higher doses of inhaled anesthetics when compared to dark-haired people [2]. Red hair is associated with mutations of the human melanocortin 1 receptor (MC1R), and deficiency of MC1R signaling is characterized by reduced availability of NO [3].

Nitric oxide (NO) is a neurotransmitter in the central nervous system that modulates signaling pathways relevant to general anesthesia, including those involving GABA [4], NMDA [5], and acetylcholine [6]. NO is enzymatically generated by NO synthase (NOS) and can signal through a multitude of downstream targets, including guanylyl cyclase (GC). Upon activation by NO, GC, a cytosolic heme protein consisting of an α1 or α2 subunit combined with a common β1 subunit, produces the second messenger cyclic guanosine monophosphate (cGMP) [7]. Although GC-1 is the predominant isoform throughout most organ systems including the cardiovascular system, similar levels of GC-1 and GC-2 are expressed in the brain [8]. In the central nervous system, GC behaves like a neurotransmitter receptor characterized by rapid activation and slow desensitization [9]. The ability of cGMP to control synaptic plasticity in the mammalian brain is well documented [10]. Downstream targets of cGMP include cGMP-dependent protein kinases (PKG), phosphodiesterases, and cGMP-gated ion channels.

Animal models are useful to explore the mechanisms whereby the NO-cGMP system produces volatile anesthesia’s effects on the brain. For example, halothane [11–15], isoflurane [12, 16–18] and sevoflurane [12, 16] each increase NO levels [11, 12, 16, 17] in the brain that were abrogated by administration of NOS inhibitors [12, 17]. These anesthetics increase NO metabolites (nitrite and nitrate) [18], and cGMP levels [13–15]. The γ-aminobutyric acid type A (GABA_A_) receptor, an important molecular target for general anesthetics, modulates NO-mediated cGMP synthesis in the rat cerebral cortex *in vivo* [19]. A higher threshold for ethanol-induced loss of righting reflex (LORR) was reported in a murine model of PKG deficiency [20], suggesting that NO-cGMP-PKG impacts ethanol-induced hypnosis. However, the precise role of NO-cGMP signaling in the hypnotic action of volatile anesthetics remains incompletely understood.

In a previous study of cardiac ischemia-reperfusion injury in mice deficient in GC-1α (GC-1^−/−^) [21], we noted that higher concentrations of isoflurane were required to anesthetize GC-1^−/−^ mice than wild type (WT) mice (unpublished observations). In the current report, we sought to examine the impact of congenital GC-1 deficiency on the sensitivity to sevoflurane-induced unconsciousness, by measuring the LORR and return of the righting reflex (RORR) of mice [22]. We hypothesized that activation of GC-cGMP contributes to the hypnotic effect of sevoflurane, and that GC-1^−/−^ mice would be less sensitive to sevoflurane-induced hypnosis.

## Methods

### Experimental animals

All experimental animal protocols were approved by the Subcommittee on Research Animal Care at Massachusetts General Hospital (The Institutional Animal Care and Use Committee #2010 N000098), which conforms to the Guide for the Care and Use of Laboratory Animals published by the National Institutes of Health [23], and all experiments also conformed to Belgian and European law on laboratory animal experimentation and were approved by the Local Ethical Committee of Ghent University (#99/26).

GC-1^−/−^ mice with a targeted deletion of exon 6 of GC-1α were generated on a 129S6 background (Taconic, Hudson, NY), as previously described [24]. Body weight- and age-matched WT 129S6 (Taconic, Hudson, NY) and GC-1^−/−^ mice were studied. The demographics of mice (numbers, body weights, and ages) are presented in Table 1 and were similar between WT and GC-1^−/−^ mice. A 12-h dark-light cycle beginning at 7:00 am (lights on) was maintained in murine housing, with water and a normal diet provided ad libitum. All experiments were performed between 9:00 am and 3:00 pm.

**Table 1:** Numbers, body weights and ages of mice used for the LORR and RORR experiments

	Female WT	Female GC-1−/−	P	Male WT	Male GC-1−/−	P
# of mice	8	8		6	6	
BW (grams)	19.7 (18.6-20.7)	19.3 (18.8-19.9)	0.2656	24.7 (23.0-26.4)	24.7 (22.2-27.2)	0.8983
Age (weeks)	11.5 (10-12)	13.3 (12-14)	0.0002	12.1 (12-13)	12.6 (10-15)	0.5628

Data are presented as mean (95% CI)

### Loss and return of the righting reflex

Anesthetic threshold was assessed behaviorally by measuring the LORR and RORR. Male but not female GC-1^−/−^ mice have systemic hypertension [24], and female hormones can modulate cognitive function [25] and alter GC activity in the hypothalamus [26]. Therefore, LORR and RORR were reported for each gender separately, as well as both genders combined. Unrestrained, sevoflurane naive mice were placed in individual wire-mesh cages rotating at 4 rpm in a 14 l glass chamber (Chamber A) [22]. Ten liters per minute (l/min) of oxygen was used to deliver sevoflurane (Piramal Critical Care, Inc., Bethlehem, PA). Sevoflurane and oxygen concentrations inside Chamber A were measured at a gas sampling flow rate of 200 ml/min, with an infrared gas analyzer for sevoflurane and a paramagnetic oxygen analyzer (Datascope Gas Module II, with anesthesia monitor Passport 2, Mindray DS USA, Inc., Mahwah, NJ). The tip of the gas sampling tube was secured at the level of the median height of the mesh rotation cage within Chamber A. Mice were habituated to the mesh rotation cage while inhaling room air inside Chamber A, rotating at 4 rpm for 2 h/day on 4 consecutive days prior to the day of an anesthesia experiment. During experiments, controlled concentrations of sevoflurane were delivered to mice with a temperature-compensated vaporizer (Baxter Drager Vapor 2000, Drägerwerk AG & Co. KGaA, Lübeck, Germany) in 100% oxygen. Investigators blinded to the genotype of the mice observed the mice continuously, and recorded the timing of LORR and RORR onset and the sevoflurane concentrations required for LORR and RORR for each mouse.

**Fig. 1:**
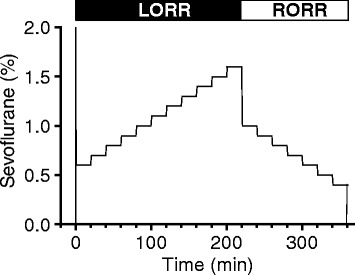
Induction (loss of righting reflex, LORR) and emergence (return of righting reflex, RORR) protocol for mice receiving sevoflurane. During the induction phase, the chamber sevoflurane concentration was increased to 2% for 30 s then rapidly decreased to 0.6%. Next, the sevoflurane dose was increased stepwise (0.1% every 20 min) up to 1.6%, then decreased to 1.0% by stepwise decreases (0.1% every 20 min) until all mice regained the righting reflex. The total duration of the experiment was 360 min, with an induction phase of 220 min and an emergence phase of 140 min

*Induction phase:* Two mice, one WT and one GC-1^−/−^ mouse, were placed in separate mesh rotation cages, and rotated inside Chamber A. On the day of the experiment, mice were habituated for 30 min at 4 rpm inhaling 10 l/min O_2_ without sevoflurane. Subsequently, a high concentration of sevoflurane was delivered briefly (2% for 30 s), and then the delivered sevoflurane concentration was decreased to maintain a concentration of 0.6% in the chamber. The concentration in the chamber was kept constant for 20 min, followed by stepwise increases in the sevoflurane concentration by 0.1% increments, up to the highest concentration of 1.6% (Fig. 1).

*Emergence phase*: Thereafter, the sevoflurane concentration was decreased from 1.6% to 1.0% (by 0.1% decrements) until all mice regained the righting reflex (Fig. 1). Each sevoflurane concentration was maintained for 20 min to allow the anesthetic dose to reach a steady state in the brain.

We defined LORR as the inability of the mouse to maintain itself on all four feet, and RORR as the time when a mouse was able to right itself again for the first time. If a mouse lost or regained its righting reflex during the first 5 min after increasing or decreasing the sevoflurane concentration, the sevoflurane concentration required for LORR and RORR was calculated by (previous {sevo} + current {sevo}), divided by 2.

Since general anesthesia impairs thermoregulation, ambient temperature inside Chamber A was recorded every 30 s (Thermometer #90121, Springfield Precision, Wood-Ridge NJ). A separate set of experiments was carried out to evaluate the impact of prolonged exposure to sevoflurane on core body temperature. Body temperature was measured with a rectal probe at the end of the induction period when mice were maximally anesthetized (220 min after initial exposure to sevoflurane with the highest sevoflurane concentration of 1.6%). During this time, the ambient temperature inside Chamber A was maintained between 27.5-29.0 **°**C using a heating lamp. Under the same experimental conditions, we confirmed that the rectal temperatures of WT and GC-1^−/−^ mice were 39 ± 1.0 and 39 ± 0.8 °C, respectively (*n* = 7 each, *p* = 0.7769) at 0 min, and 37 ± 0.5 and 37 ± 0.7 °C (*n* = 7 each, *p* = 0.4986) at 220 min.

### In situ hybridization for GC-1α and GC-2α in mouse brain

Probes specific for the GC-1α and GC-2α isoforms were cloned in vectors flanked by SP6 and T7 or T3 and T7 promoters, respectively. The GC-1α-specific probe was 290 base pair (bp) long, spanning a cDNA sequence from exons 2-4, while the GC-2α-specific probe was 170 bp and contained the cDNA sequence from exon 8. In vitro transcription of these probes using SP6, T3, or T7 polymerase (Riboprobe, Promega) with digoxigenin-labeled UTP was performed to generate labeled sense and antisense transcripts. Non-incorporated nucleotides were removed by gel filtration after in vitro transcription. The specificity of the respective in situ probes was tested on a dot blot using spotted dilutions of GC-1α, GC-2α, and GC-1β cDNA. This blot showed that each probe is isoform-specific. The GC-1α-specific probe was 290 bp long, spanning cDNA sequence from exons 2-4, while the GC-2α-specific probe was 170 bp and contained cDNA sequence from exon 8, and both regions were chosen because of their dissimilarity between isoforms. The 290 bp GC-1α-specific cDNA fragment is only 44% identical to GC-2α cDNA, and the GC-2α probe is only 51% identical to GC-1α, both containing many gaps in the sequence alignment.

Male WT mice were deeply anesthetized with tribromoethanol (intraperitoneal, 250 mg/kg), the chest was opened, the inferior vena cava was cut, and mice were perfused first with PBS and then with 4% paraformaldehyde (PFA) in PBS via infusion into the left ventricle. Next, whole mouse brains were dissected and immediately fixed in 4% PFA in PBS for 12 h at 4 °C, and then embedded in paraffin. Six-μM thick sections, cut with a semi-automated microtome (Leica), were probed for the presence of GC-1α and GC-2α mRNA using standard in situ hybridization techniques. In brief, after deparaffination and dehydration sections were briefly post-fixed in 4% PFA and blocked in 0.1 M glycine. Permeabilization with 10% proteinase K was followed by a second post-fixation step, and prehybridization at 55 °C for 20 min in hybridization solution containing 0.5 mg/ml tRNA, 0.5 mg/ml salmon sperm DNA, 1.25× SSC, 30% formamide, and 0.25× Denhardt’s solution. Consecutive sections were then incubated overnight at 55 °C with digoxigenin-labeled antisense and sense probes, followed by washing in 2xSSC and 1xSSC at 40 °C. After blocking the section in sheep serum, the digoxigenin label was detected using anti-digoxigenin-antibody coupled to alkaline phosphatase (Roche). Finally, sections were developed in NBT/BCIP solution to visualize GC-1α- and GC-2α-specific staining. Staining of sections with sense probes was used as negative controls. Anatomic regional localization of GC-1α- and GC-2α-positive cells were confirmed by comparisons with the Allen mouse brain atlas map of genes [27].

### Brain cGMP measurements

Mice were habituated to being restrained in tapered plastic cones (a 140 cm^3^ cone; Mouse Decapicone, DC-M200, Braintree Scientific, Braintree, MA) for 20 min, twice daily for three consecutive days prior to the day of study. In order to obtain accurate brain cGMP analyses, mice received an intraperitoneal injection of the phosphodiesterase inhibitor 3-isobutyl-1-methylxanthine (IBMX, 0.01 mcg/g, I-5879, Sigma-Aldrich, St. Louis, MO) [28], right before going into a 2.5 l volume acrylic anesthesia induction chamber (10″ × 4″ × 4″, Gas Anesthetizing Box, AB-1, Braintree Scientific); Chamber B.

Mice breathed oxygen (5 l/min) without or with 1.2% sevoflurane (Dräger Vapor 2000,Telford, PA) in Chamber B, for 20 min. Rectal temperature was continuously monitored (Mouse Rectal Temperature Probe, 382-0001-01, Indus Instruments, Webster, TX, Traceable 2-Channel Thermometer, VWR International, Radnor, PA) and maintained at 36.5-37.5 °C with a heating lamp. Concentrations of sevoflurane and oxygen inside Chamber B were sampled and continuously measured with a gas analyzer (Datascope Gas Module II-Passport 2 system).

After 20 min of inhaling oxygen with/without sevoflurane, mice were decapitated with a guillotine (Nemi Guillotine, NS-80-1, Braintree Scientific) immediately after removal from the chamber. To prevent degradation of cGMP, dry ice was applied to the Nemi Guillotine and plastic tubes, and a cold plate (Z176664, Sigma-Aldrich) was used while dissecting the brain. Brain tissue was snap frozen in liquid nitrogen and stored at −80 °C until the analysis.

Brain cGMP levels were measured by blinded investigators using an ELISA [29]. Briefly, harvested organs were first powdered at −70 °C and subsequently homogenized in 1 ml ice-cold 100% ethanol. Extracts were centrifuged at 14,000 g for 10 min at 37 °C. The supernatant was transferred and the pellet was washed once with 0.5 ml ice-cold 100% ethanol and centrifuged at 14,000 g for 10 min at 37 °C. Next, the supernatant was dried under a vacuum at 30 °C. The pellet was redissolved in protein buffer (20 mmol l^−1^ HEPES, 350 mmol l^−1^ NaCl, 0.5 mmol l^−1^ EDTA, 20% glycerol, 0.5% Triton X-100, EDTA-free protease inhibitor mix), centrifuged for 10 min at 14,000 g at 37 °C, and the protein concentration was measured using a BCA Protein Assay Kit (Pierce). cGMP pellets were dissolved in EIA-buffer and cGMP concentrations were measured using the acetylation protocol of the cyclic GMP EIA Kit (Cayman Chemical). cGMP concentration is expressed as picomoles cGMP per milligram of brain protein. For all cGMP experiments, mice were enrolled in small cohorts (e.g. 2-4).

### Data acquisition and statistical analysis

All data are presented as mean ± standard deviation. Sample sizes were obtained by statistical analysis software G*Power (G*Power 3.1 [30], Heinrich-Heine-Universität Düsseldorf, Düsseldorf, Germany). Randomization methods were not used to assign mice to their experimental condition.

Power analysis: Sample sizes for LORR/RORR experiments in female mice were calculated based on our preliminary data for the two-tailed t-test: effect size d = 1.6, α error probability of 0.05 and power of 0.8. Sample size for each female group (WT, GC-1^−/−^) was 8 mice. Sample sizes for male mice were calculated based on the female LORR (complete, %) data, for the two-tailed t-test: effect size d = 2, α error probability of 0.05 and power of 0.8. Sample size for each male group (WT, GC-1^−/−^) was 6 mice. Sample sizes for the cGMP measurements were initially calculated based on estimated mean and standard deviation for the two-tailed t-test with d = 1.2, α error probability of 0.05, and power of 0.8.

To achieve this level of power, sample size for each group was required to be 12 mice. The statistical analyses for the hypothesis were conducted sequentially using small cohorts of mice (e.g. *n* = 2-4). When the sample size reached a total of *n* = 15 for the GC-1^−/−^ mice, an analysis was conducted that failed to reject the null hypothesis and demonstrated a near zero effect size, so the study was terminated without enrolling further animals. No statistical adjustments were made to account for this interim analysis.

All data were tested for normal distribution by the Shapiro-Wilk normality test. To compare LORR and RORR between WT and GC-1^−/−^, an un-paired independent t-test was used for normally distributed data, and a Mann-Whitney U test was used for parameters without a normal distribution. For comparisons of cumulative probability for LORR and RORR curves, the log rank Mantel-Cox test was used. For brain cGMP measurements, parameters were logarithmically transformed for analysis by ANOVA with Bonferroni corrections, due to their abnormal distributions. *P* values <0.05 were considered significant. All statistical analysis was performed using GraphPad Prism 6, version 5.01; GraphPad Software, La Jolla, CA.

## Results

### The sevoflurane concentration required for LORR is higher in GC-1^−/−^ mice of both genders compared to WT mice

**Fig. 2:**
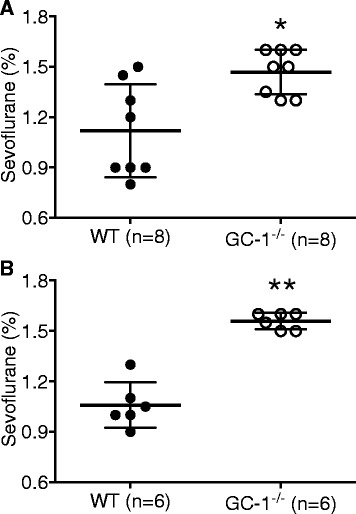
Loss of righting reflex. GC-1^−/−^ mice exhibit sevoflurane hyposensitivity relative to wild-type mice (WT) in both females (**a**) and males (**b**). Effects of genotype on LORR are significant in both genders. Data presented as mean ± standard deviation. **a**: * *P* = 0.0095, vs., WT mice. **b**: ** *P* = 0.0022, vs., WT mice

LORR and RORR are well-established endpoints to determine anesthetic-induced unconsciousness and recovery of consciousness in mice [22]. In female mice, the mean sevoflurane concentration required to induce LORR was 1.5% in GC-1^−/−^ mice (95% confidence interval; 95% CI: 1.4 to 1.6%, *n* = 8) and 1.1% in WT mice (95% CI: 0.9 to 1.4%, *n* = 8, *P* = 0.0095. Figure 2a). Similarly, in male mice, the mean sevoflurane concentration required to induce LORR was 1.6% in GC-1^−/−^ mice (95% CI: 1.5 to 1.6%, *n* = 6) and 1.1% in WT mice (95% CI: 0.9 to 1.2%, *n* = 6; *P* = 0.0022. Figure 2b). When combined, LORR occurred at higher mean sevoflurane concentrations in GC-1^−/−^ mice than in WT mice (1.5%, 95% CI: 1.4 to 1.6%, vs. 1.1%, 95% CI: 1.0 to 1.2%, respectively, *n* = 14 and 14, *P* < 0.0001).

### RORR occurred at a higher sevoflurane concentration in GC-1^−/−^ mice of both genders in comparison with WT mice

**Fig. 3:**
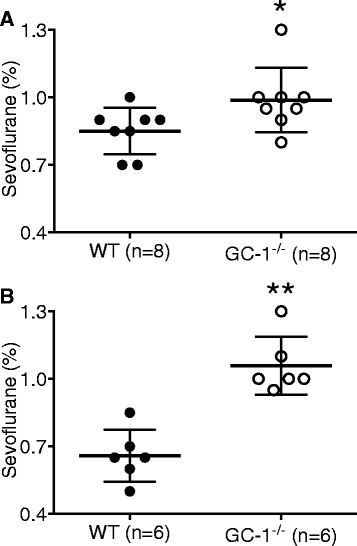
Return of righting reflex. GC-1^−/−^ mice exhibit sevoflurane hyposensitivity relative to wild-type mice (WT) in both females (**a**) and males (**b**). Effects of genotype on RORR are significant in both genders. Data presented as mean ± standard deviation. **a**: * *P* = 0.0451, vs., WT mice. **b**: ** *P* = 0.0022, vs., WT mice

In female mice, the mean sevoflurane concentrations at which RORR occurred were 1.0% in GC-1^−/−^ mice (95% CI: 0.9 to 1.1%, *n* = 8) and 0.9% in WT mice (95% CI: 0.8 to 0.9%, *n* = 8; *P* = 0.0451. Figure 3a). Similarly, in male mice, the mean sevoflurane concentration required to induce RORR was 1.1% in GC-1^−/−^ mice (95% CI: 0.9 to 1.2%, *n* = 6) and 0.7% in WT mice (95% CI: 0.5 to 0.8%, *n* = 6; *P* = 0.0022. Figure 3b). When combined, RORR occurred at higher sevoflurane concentrations in GC-1^−/−^ mice than in WT mice (1.0%, 95% CI: 0.9 to 1.1%, vs. 0.8%, 95% CI: 0.7 to 0.9%, respectively, *n* = 14 and 14, *P* < 0.0001).

Taken together, these results illustrate that GC-1^−/−^ mice of both sexes are more resistant to the hypnotic effects of sevoflurane than WT mice. Since the results were similar in both sexes, subsequent in vitro brain GC localization and cGMP measurements were obtained in female mice only.

### GC-1α and GC-2α are expressed in the cerebral cortex, medial habenula, hippocampus and cerebellum of mice

**Fig. 4:**
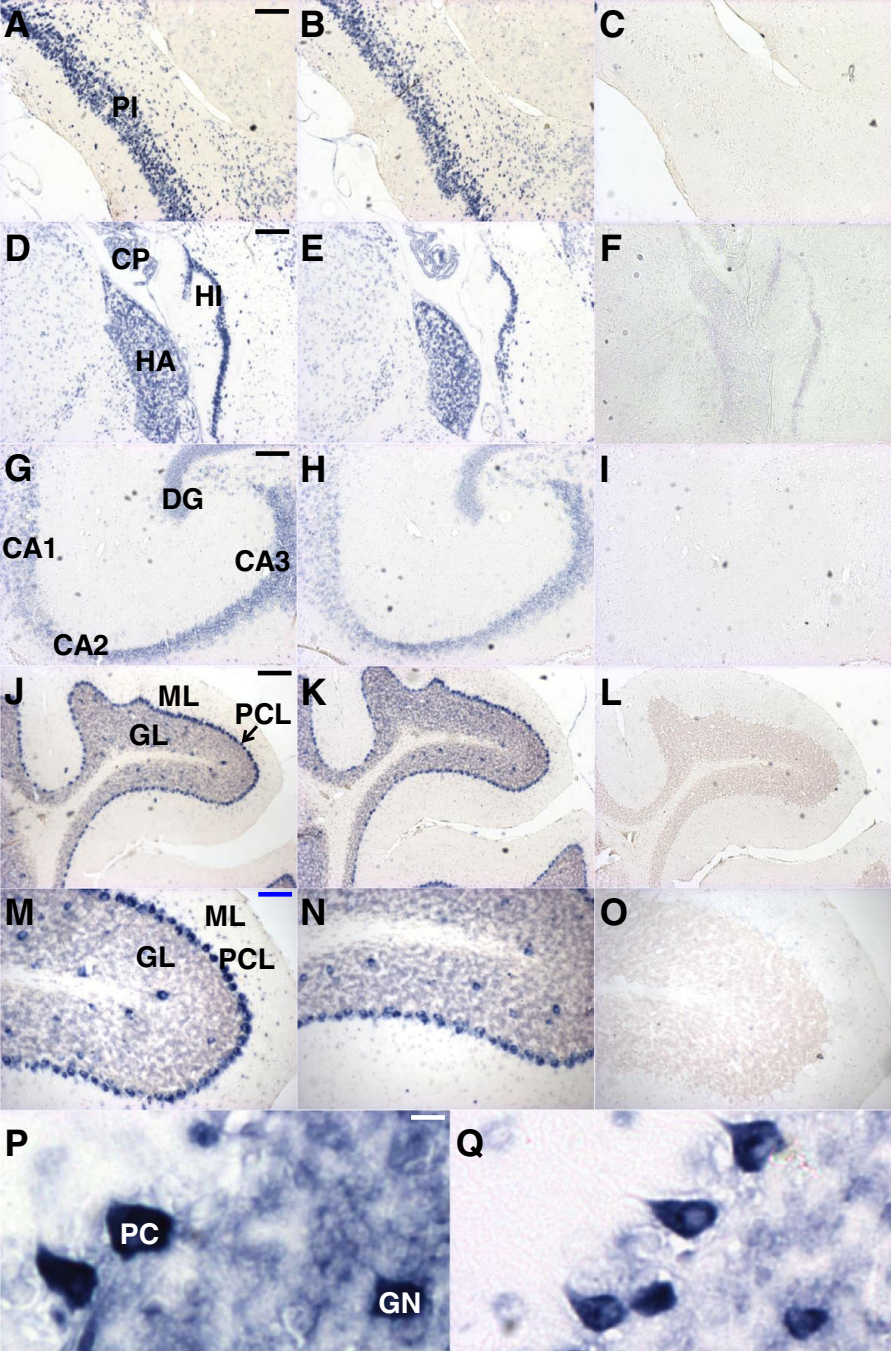
Localization of the expression of GCα isoforms in murine brain. In situ hybridization was performed on sections of adult mouse brain using GC-1α- (**a**, **d**, **g**, **j**, **m** and **p**) and GC-2α- (**b**, **e**, **h**, **k**, **n** and **q**) specific antisense probes, as well as sense probes (**c**, **f**, **i**, **l** and **o**) as a negative control (results obtained with the GC-2α sense probe were similar to those obtained with the GC-1α sense probe and are not shown here). Representative sections of the cerebrum (coronal sections of the cerebral cortex: panels **a**-**c**, sagittal view of part of the limbic system: panels **d**-**f**, saggital view of hippocampus: panels **g**-**i**) and the cerebellum (**j**-**q**) are shown. GC-1α and GC-2α were expressed in cerebral cortex neurons (layer II/III and the pyramidal layer of the piriform cortex), third ventricle of the brain (epithelial cells of the choroid plexus), thalamus (medial habenulae), hippocampus (granular cell layer of the dentate gyrus and in the pyramidal layer of the cornu ammonis), and cerebellum (Purkinje cell layer and Golgi cells of the granular layer, with lower levels detectable in granule cells). GL: granular layer, ML: molecular layer, PCL: Purkinje cell layer, GN: Golgi neuron, PC: Purkinje cell, CA1-3: pyramidal layer of regions 1-3 of the hippocampal cornu ammonis, DG: hippocampal dentate gyrus, CP: choroid plexus, HA: medial habenula, HI: hippocampus. Scale bars: black: 200 μm, blue: 80 μm, white: 10 μm

In situ hybridization using GC-1α- and GC-2α-specific antisense RNA probes revealed expression of both GC isoforms throughout the murine brain. GC-1α and GC-2α were expressed in cerebral cortex neurons, particularly in layer II/III, and in the pyramidal layer of the piriform cortex. In the third ventricle of the brain, GC mRNA was detected in epithelial cells of the choroid plexus. In the thalamus, GC-1α and GC-2α were readily detectable in the medial habenulae. In the hippocampus, high levels of GC expression were detected in the granular cell layer of the dentate gyrus and in the pyramidal layer of the cornu ammonis (CA). Staining for both isoforms was observed in the Purkinje cell layer and Golgi cells of the granular layer of the cerebellum, with lower levels detectable in granule cells (Fig. 4).

### *Sevoflurane increases whole brain cGMP in WT but not in* GC-1^−/−^ mice

**Fig. 5:**
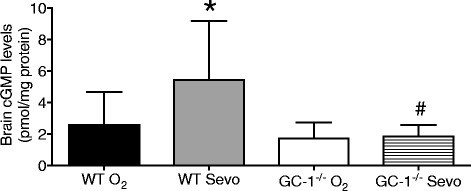
Sevoflurane increases brain cGMP levels in WT mice but not in GC-1^−/−^ mice. Brain cGMP levels in wild-type mice (WT) and GC-1^−/−^ mice inhaling 1.2% sevoflurane for 20 min. The control group (O_2_) breathed O_2_. Sevoflurane (Sevo) increased whole brain cGMP levels in WT mice, but not in GC-1^−/−^ mice. Data presented as mean ± standard deviation. Adjusted *P* values: * *P* = 0.0355 vs. WT O_2_ and *P* = 0.0062 vs. GC-1^−/−^ O_2_, ^#^ *P* = 0.0275 vs. WT Sevo, by one-way ANOVA. *N* = 13, 10, 8, 7 in WT O_2_, WT Sevo, GC-1^−/−^ O_2_, GC-1^−/−^ Sevo, respectively

To test whether GC-1 is involved in the sevoflurane-induced cGMP increase in the brain, female WT and GC-1^−/−^ mice inhaled oxygen with or without 1.2% sevoflurane for 20 min. cGMP levels were measured in the whole brain. In WT mice, inhalation of 1.2% sevoflurane increased the levels of cGMP in the brain: average cGMP levels were 2.6 pmol/mg protein (95% CI: 1.4 to 3.9, *n* = 13) and 5.5 pmol/mg protein (95% CI: 2.8 to 8.1, *P* = 0.0355, vs. O_2_ alone, *n* = 10) in mice inhaling O_2_ alone and 1.2% sevoflurane in O_2_, respectively (Fig. 5). In contrast, cGMP levels were similar in the brain of GC-1^−/−^ mice inhaling O_2_ alone (1.8 pmol/mg protein, 95% CI: 1.0 to 2.6, *n* = 8) or 1.2% sevoflurane in O_2_ (1.9 pmol/mg protein, 95% CI: 1.3 to 2.5, *n* = 7, *P* = 0.9724, Fig. 5). These results provide evidence that sevoflurane increases cGMP levels in the whole brain in an GC-1-dependent manner.

## Discussion

In this study, we report that a congenital deletion of GC-1α increases the concentration of sevoflurane required to produce LORR in mice of both sexes. Expression of both GC-α isoforms (α1 and α2) was detected in the cerebral cortex, medial habenulae, cerebellum and hippocampus. During sevoflurane inhalation, levels of cGMP in the whole brain were increased in WT mice, but not in GC-1^−/−^ mice. These results highlight a significant role for the NO-GC-cGMP pathway in modulating sevoflurane-induced unconsciousness.

In the current study, LORR occurred at a higher concentration of sevoflurane in mice of both sexes with a congenital GC deficiency as compared to WT mice, indicating an important role for brain GC levels in loss of consciousness and regaining wakefulness. One of the major molecular targets for sevoflurane is thought to be the GABA_A_ receptor in the cortex [31], and the NO-cGMP signaling system is present and active in cortical GABA neurons. For example, cortical GABA_A_ neurons modulate NO-mediated cGMP synthesis [19], and the heme-dependent GC stimulators BAY 41-2272 and YC-1 potentiate cGMP synthesis upon stimulation by NO-donors in GABAergic and glutamatergic neurons in the cortex [32]. Moreover, inhibition of the GABA_A_ receptor by the selective GABA_A_ receptor antagonist bicuculline increased cortical cGMP production in rats. This increased cGMP production in response to bicuculline may be indicative of a negative feedback mechanism to further stimulate the GABA receptor [19]. The exact mechanisms behind cGMP’s direct effects on the cortical GABA_A_ neurons remains to be elucidated.

We detected both GC-1α and GC-2α positive cells in the cerebral cortex, medial habenulae, hippocampus, cerebellum, and epithelial cells of the choroid plexus. Our results are in accordance with the Allen mouse brain atlas map of genes [27] and concur with previous reports of the distribution of GC in the rat brain [33, 34]. cGMP binding sites were identified in regions overlapping with, or adjacent to, GC positive regions including the habenulae, hippocampus, and cerebellum [35, 36], illustrating the presence of a fully functional cGMP signaling system in areas of the central nervous system that may regulate susceptibility to anesthesia. For example, the strongest in situ hybridization signals for both GC-1α and GC-2α were found in the medial habenula, both in the current study as well as by others [36]. GC co-localizes with cGMP receptors in the medial habenulae [35]. The medial habenulae have been reported to regulate pain, anxiety, and sleep [37, 38], and general anesthesia increased glucose uptake (an indicator of the brain’s metabolic activity) in the habenular system [39]. GC-cGMP signaling in the medial habenulae may play an important role in modulating murine sensitivity to anesthesia.

The role of NO-cGMP signaling in analgesia and loss of consciousness induced by general anesthesia has been a focus of intense investigation. Possible mechanisms exerted by cGMP on brain function have been thoroughly reviewed, specifically in the context of neuronal long-term potentiation or depression in hippocampus, amygdala and cerebellum to exert complex behaviors [10]. cGMP increases NMDA receptor currents via HCN channels contributing in both pre- [40] and post- [41] synaptic plasticities. In vivo, the majority of studies reported that pharmacological inhibition of NOS, either all NOS enzymes non-selectively or NOS1 [22, 42–45], of GC [46, 47], or of PKG-Iα [48] *reduces* MAC and/or LORR in rodents. While these results differ from the current observations, pharmacological inhibitors are often nonspecific and may have “off-target” effects. It has also been suggested that acute and chronic inhibition of NO-cGMP signaling can produce distinct and differing effects on anesthetic sensitivity [22, 44]. Therefore, a life-long congenital deficiency of NOS or GC vs. acute pharmacological inhibition may have different effects on anesthetic sensitivity.

The current study provides the first evidence that congenital GC-1α deficiency decreases anesthetic sensitivity to sevoflurane in mice using proper genetic controls. Two studies reported the impact of congenital NOS1 (neuronal NOS) deficiency on anesthetic sensitivity. Ichinose and colleagues reported that NOS1 deficiency did not affect minimum alveolar concentration (MAC) and LORR to isoflurane anesthesia [22] whereas Engelhardt and colleagues showed that NOS1 deficiency markedly *increased* isoflurane MAC and tended to increase the required isoflurane concentration for LORR in mice [45], in line with the current observations. The variable effects of NOS1 deficiency on anesthetic sensitivity may be attributable to differences in the genetic background of the mice that were studied, possibly associated with the presence of additional genetic modifiers. It has been reported that different mouse strains exhibit markedly altered anesthetic sensitivity [49]. Both the GC-1^−/−^ mice and WT mice we studied were on the 129S6 background, while NOS1^−/−^ and WT mice studied by Ichinose et al. and Engelhardt and colleagues were on a mixed B6/129 backgrounds. Also, it is important to note that NO has targets beyond GC, and that differences observed in NOS-deficient mice may be attributed to NOS-dependent but GC-independent effects.

In humans, several families have been identified that carry mutations in the gene that encodes GC-1α (GUCY1A3) or the GC stabilizer (CCT7) [50, 51], resulting in a loss of GC activity. Subjects carrying these mutations are highly susceptible to develop Moyamoya disease [50], achalasia [50] and myocardial infarction [51]. Anesthetic requirements for patients with GC mutations, and the precise mechanisms for increased anesthesia requirements of some patients remain unclear. Our data suggest that impaired NO-cGMP signaling, due to genetic mutations or other biochemical abnormalities (e.g. decreased NO bioavailability), may impact the doses of inhaled anesthetics required to induce unconsciousness. For example, anesthetic requirements were reported to be increased in humans with red hair [2], carrying mutations in the melanocortin-1 receptor (MC1R) gene [2, 52]. These genetic variants were previously reported to modulate κ-opioid analgesia in humans [53]. More recently, however, results from a matched-cohort study revealed that red-hair phenotype does not appear to alter risk of intraoperative awareness and postoperative recall, anesthetic requirements, or recovery characteristics in surgical patients [54].

Since reduced endothelial NOS expression and decreased plasma levels of NO metabolites were described in mice deficient in MC1R signaling [3], it remains conceivable that downregulation of NO-cGMP signaling induced by dysfunctional MC1R contributes to the decreased sensitivity to inhaled anesthetics patients with dysfunctional MC1R gene. Future investigations are required to further understand how NO-cGMP affects the threshold of anesthesia.

In summary, we report that GC-1^−/−^ mice require higher doses of sevoflurane to achieve hypnosis compared to WT mice. Although sevoflurane increased brain cGMP levels in WT mice, this change was not observed in GC-1^−/−^ mice. These findings encourage additional studies to elucidate how the NO-GC-cGMP pathway is involved in anesthetic-induced hypnosis.

## Conclusions

We report that congenital GC-1 deficiency decreases the sensitivity to sevoflurane anesthesia in mice of both sexes. The sevoflurane-induced increase in cGMP in the WT brain was abrogated in mice congenitally lacking GC-1. Our results suggest that patients with impaired NO-cGMP signaling secondary to genetic mutations affecting the NO-cGMP system may require higher anesthetic concentrations to induce unconsciousness.

## Abbreviations

cGMP: cyclic guanosine monophosphate
GABA_A_: γ-aminobutyric acid type A
GC: Guanylyl cyclase
GC-1^−/−^: mice congenitally deficient in GC-1α
GC-1α: α1 subunit of GC
GC-1β: β1 subunit of GC
GC-2α: α2 subunit of GC
IBMX: 3-isobutyl-1-methylxanthine
LORR: Loss of righting reflex
MAC: Minimum alveolar concentration
MC1R: Melanocortin 1 receptor
NO: Nitric oxide
NOS: NO synthase
PFA: Paraformaldehyde
PKG: cGMP-dependent protein kinases
RORR: Return of righting reflex
WT: Wild-type mice
